# Prognostic value of angiographic microvascular resistance in patients with ST-segment elevation myocardial infarction

**DOI:** 10.1016/j.clinsp.2024.100429

**Published:** 2024-07-24

**Authors:** Gangzhen Qian, Haoran Qin, Dan Deng, Yue Feng, Chao Zhang, Xiaolong Qu, Zhihui Zhang

**Affiliations:** Department of Cardiovascular Medicine, Center for Circadian Metabolism and Cardiovascular Disease, Southwest Hospital, Army Medical University, Chongqing, China

**Keywords:** ST-segment elevation myocardial infarction, AMR, Coronary physiology, Coronary angiography, Prognosis

## Abstract

**Background:**

The Angiographic Microvascular Resistance (AMR), derived from a solitary angiographic view, has emerged as a viable substitute for the Index of Microcirculatory Resistance (IMR). However, the prognostic significance in ST-Segment Elevation Myocardial Infarction (STEMI) patients is yet to be established. This research endeavors to explore the prognostic capabilities of AMR in patients diagnosed with STEMI.

**Methods:**

In this single-center, retrospective study, 232 patients diagnosed with STEMI who received primary Percutaneous Coronary Intervention (PCI) were recruited from January 1, 2018, to June 30, 2022. Utilizing the maximally selected log-rank statistics analysis, participants were divided into two cohorts according to an AMR threshold of 2.55 mmHg*s/cm. The endpoint evaluated was a composite of all-cause mortality or hospital readmission due to heart failure.

**Results:**

At a median follow-up of 1.74 (1.07, 3.65) years, the composite endpoint event was observed in 28 patients within the higher AMR group and 8 patients within the lower AMR group. The higher AMR group showed a significantly higher risk for composite outcome compared to those within the low-AMR group (HR_adj_: 3.33; 95% CI 1.30‒8.52; p = 0.03). AMR ≥ 2.55 mmHg*s/cm was an independent predictor of the composite endpoint (HR = 2.33; 95% CI 1.04‒5.21; p = 0.04). Furthermore, a nomogram containing age, sex, left ventricle ejection fraction, post-PCI Quantitative Flow Ratio (QFR), and AMR was developed and indicated a poorer prognosis in the high-risk group for STEMI patients at 3 years. (HR=4.60; 95% CI 1.91‒11.07; p < 0.01).

**Conclusions:**

AMR measured after PCI can predict the risk of all-cause death or readmission for heart failure in patients with STEMI. AMR-involved nomograms improved predictive performance over variables alone.

## Highlights

• AMR measured after PCI can predict the risk of MACEs in patients with STEMI.
• AMR-involved nomogram improved predictive performance over variables alone.
• AAMR-involved nomogram-derived high-risk population showed a worse prognosis at 3 years.
• AMR has the potential to be a feasible alternative for IMR.

## Introduction

Cardiovascular Disease (CVD) is a well-known primary factor of mortality globally,^1^ with ST-Segment Elevation Myocardial Infarction (STEMI) representing a particularly acute manifestation of CVD, which accounts for a considerable proportion of mortality and morbidities. STEMI arises due to the rupture or erosion of unstable plaques, compounded by atherosclerosis in the Infarct-Related Artery (IRA), leading to immediate thrombus formation within the IRA. Consequently, blood flow is abruptly reduced or halted, resulting in severe acute ischemic necrosis of the myocardium supplied. It has been established that Percutaneous Coronary Intervention (PCI) is the optimal emergency management for STEMI in order to restore blood flow to the occluded IRA.^2^^,^^3^ Nonetheless, it remains critical to acknowledge that despite the prompt and effective revascularization of major epicardial arteries, Coronary Microvascular Diseases (CMD) continue to influence long-term outcomes in patients with STEMI.^4^, ^5^, ^6^

The Index of Microcirculatory Resistance (IMR) was found to be an effective, real-time tool to assess CMD in patients with coronary artery diseases by the largest proportion of clinical evidence. Generally, CMD is observed with IMR ≥ 25,^7^ whereas IMR > 40 measured after PCI may indicate risk of Major Adverse Cardiovascular Events (MACEs),^8^ such as death, readmission for heart failure, etc.

Nonetheless, the use of IMR in clinical practice, especially in urgent situations, is limited due to its dependency on hyperemia. Recent development in functional coronary angiography^9^ enables accurate and feasible measurement of coronary microcirculation among STEMI patients.

A Former study described a novel indicator, Angiographic Microvascular Resistance (AMR) which is inferred from a single angiographic view, and showed good correlation with IMR in the cohort of acute and chronic syndrome.^10^ However, there is still uncertainty about the prognostic value of the parameter among patients with STEMI after successful PCI. The study focused on evaluating the prognostic significance of AMR in patients with STEMI, as well as furnishing clinical cardiologists with practical tools for an initial risk and prognosis evaluation of STEMI, thereby mitigating unnecessary financial burdens on patients.

## Methods

### Study design

Patients who underwent emergency PCI for STEMI at the Department of Cardiovascular Medicine in the Southwest Hospital between January 1, 2018, and June 30, 2022, were consecutively recruited in this study. STEMI was confirmed based on the universal definition of myocardial infarction, as per the guidelines set by the ESC/ACC/AHA/WHF Expert Consensus Document.^11^ The exclusion criteria were: 1) Patients with STEMI who died in hospital after PCI or who were unable to receive further treatment and continuous follow-up due to patients’ reasons; 2) Patients whose coronary angiography images did not meet the requirements for AMR analysis, for example, the poor contrast opacification or severely overlapping vascular structures. AMR of the IRA was determined through computational analysis based on the final coronary angiography prior to discharge. The research received approval from the Southwest Hospital (Approval Number (B) KY2023069) on the 20^th^ of June, 2023, and was carried out respecting the guidelines set forth in the Declaration of Helsinki. The study has been carried out in accordance with the STROBE Statement. Due to the study's retrospective design, the requirement for informed consent was exempted.

### Computation of AMR

A single-view AMR and Quantitative Flow Ratio (QFR) analysis based on Murray's law was performed at the IRA after successful PCI by using the QFR software (AngioPlus Gallery, Pulse Medical Technology Inc., Shanghai, China). By applying automated processes, luminal contours of the interrogated coronary artery are meticulously delineated in the optimal angiographic perspective to minimize vessel overlap and achieve optimal clarity. An estimate of hyperemic flow velocity is calculated by dividing the vessel centerline length by the duration of contrast filling. Using an analytical frame that features complete contrast fill-in and thorough exposure of luminal contours, the vessel perimeter and major branch boundaries are automatically delineated. The step-down phenomenon exhibited across bifurcations can be used to determine the diameter of a reference vessel using Murray's bifurcation fractal law, as previously reported.^12^^,^^13^ The calculation of AMR is based on dividing the Distal Pressure (Pd) by the hyperemic flow velocity (Velocity_hyperemia_) displayed in the distal coronary arteries.

$$AMR=\frac{P_{d}}{Velocity_{hyperemia}}=\frac{estimated\;P_{a}*QFR}{Velocity_{hyperemia}}$$

The algorithm based on machine learning determines the estimated hyperemic coronary flow velocity automatically in this parameter. In our study, independent operators, who were not informed about the patients' data and clinical outcomes, conducted a blinded analysis.

### Follow-up and outcomes

The study's endpoint was the occurrence of either all-cause mortality or hospital readmission for heart failure during the follow-up period. The median duration of follow-up was 1.74 years, with a range from 1.07 to 3.65 years. Follow-up data were gathered from routine outpatient visits, reviews of medical records, and telephone communications.

In accordance with prevailing practices, all deaths are assumed to be caused by cardiac etiology unless explicit documentation indicates otherwise. Hospital readmission for heart failure is characterized by the recent emergence or worsening of cardiac insufficiency, coupled with a left ventricular Ejection Fraction (EF) less than 50% as documented by cardiac ultrasound, a noticeable increase in B-type natriuretic peptide levels, or a heart failure diagnosis at discharge. These events are confirmed by experienced cardiologists conducting blinded assessments.

### Statistical analysis

Employing the maximally selected log-rank statistics method,^14^ the optimal threshold for AMR was identified. Restricted Cubic Splines (RCS) analysis was then applied to illustrate the relationship between the AMR as a continuous variable and the risk of all-cause mortality and readmission for heart failure in patients with STEMI. Categorical variables are depicted through numerical counts and proportional occurrences, whereas continuous variables are delineated by mean values accompanied by standard deviations or median values with interquartile ranges, contingent upon their distributional characteristics as verified by Shapiro-Wilk tests.

Using a univariate Cox model, we identified AMR-related independent factors for STEMI patients' composite endpoints. Variables with p-values less than 0.05 were chosen for inclusion in a multivariate Cox analysis, which also considered age, gender, and post-PCI QFR. These were then integrated into the development of a prognostic nomogram aimed at estimating the 3-year survival probability. Subsequent analyses involved using the Area Under the Curve (AUC) in Receiver Operator Characteristic (ROC) analysis to assess and compare the effectiveness of the nomogram against the individual variables when considered separately. Kaplan Meier curves with as well as without Inverse Probability of Treatment Weighting (IPTW) adjustment were plotted to estimate survival for the composite endpoints stratified by AMR and AMR-derived nomogram (expressed as dichotomous variables). A p-value less than 0.05 is deemed statistically significant, and all probability values are two-tailed.

## Results

**Fig. 1:**
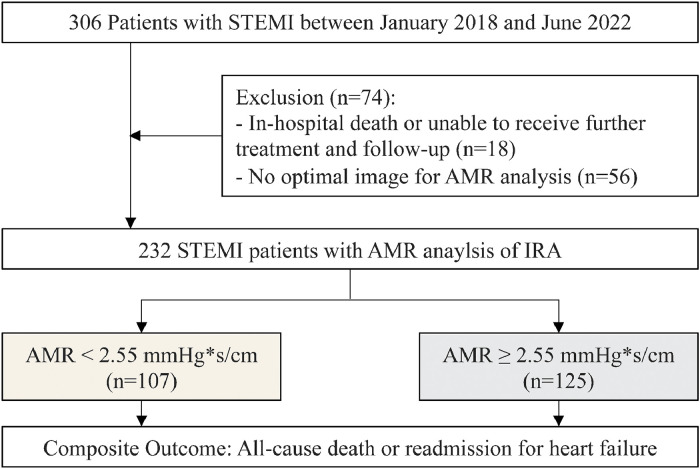
Flowchart for patient selection.

A total of 306 patients with STEMI were initially screened, and ultimately, 232 STEMI patients were selected for inclusion in the study. Fig. 1 presents a flow chart illustrating the patient selection process.

### Baseline characteristics according to AMR

**Fig. 2:**
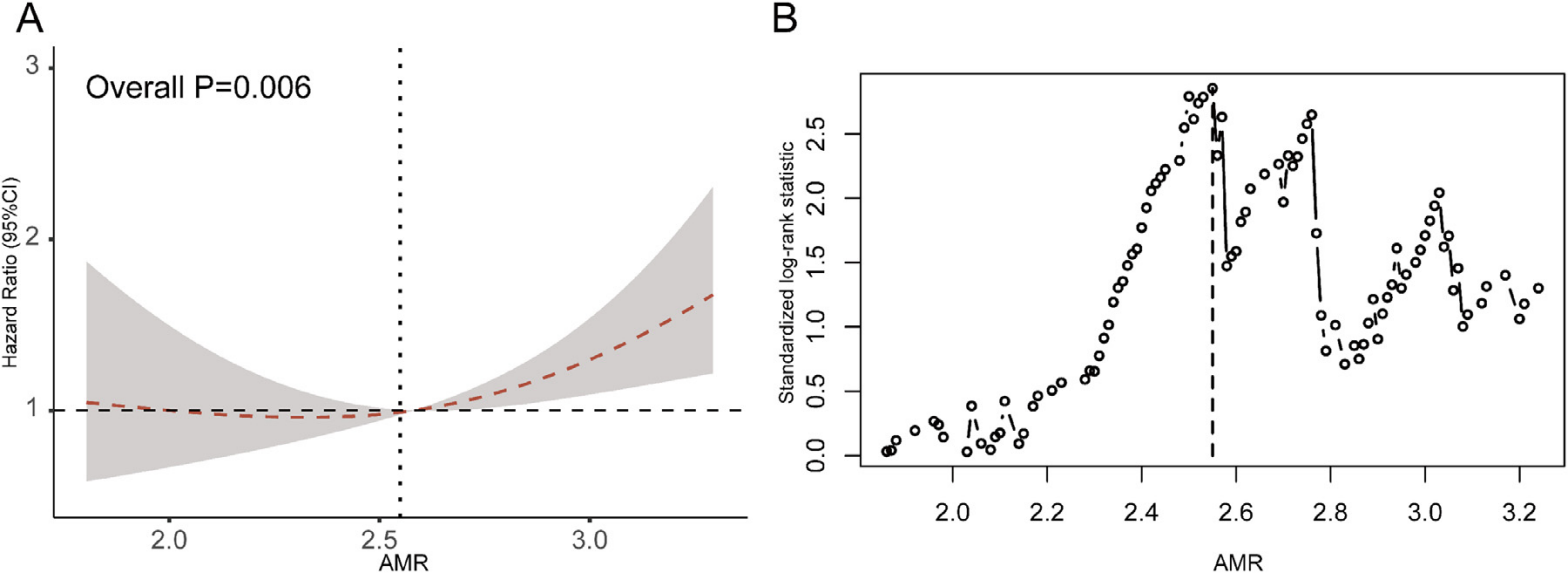
Best cut-off value of AMR for all-cause death or readmission for heart failure.

**Fig. 3:**
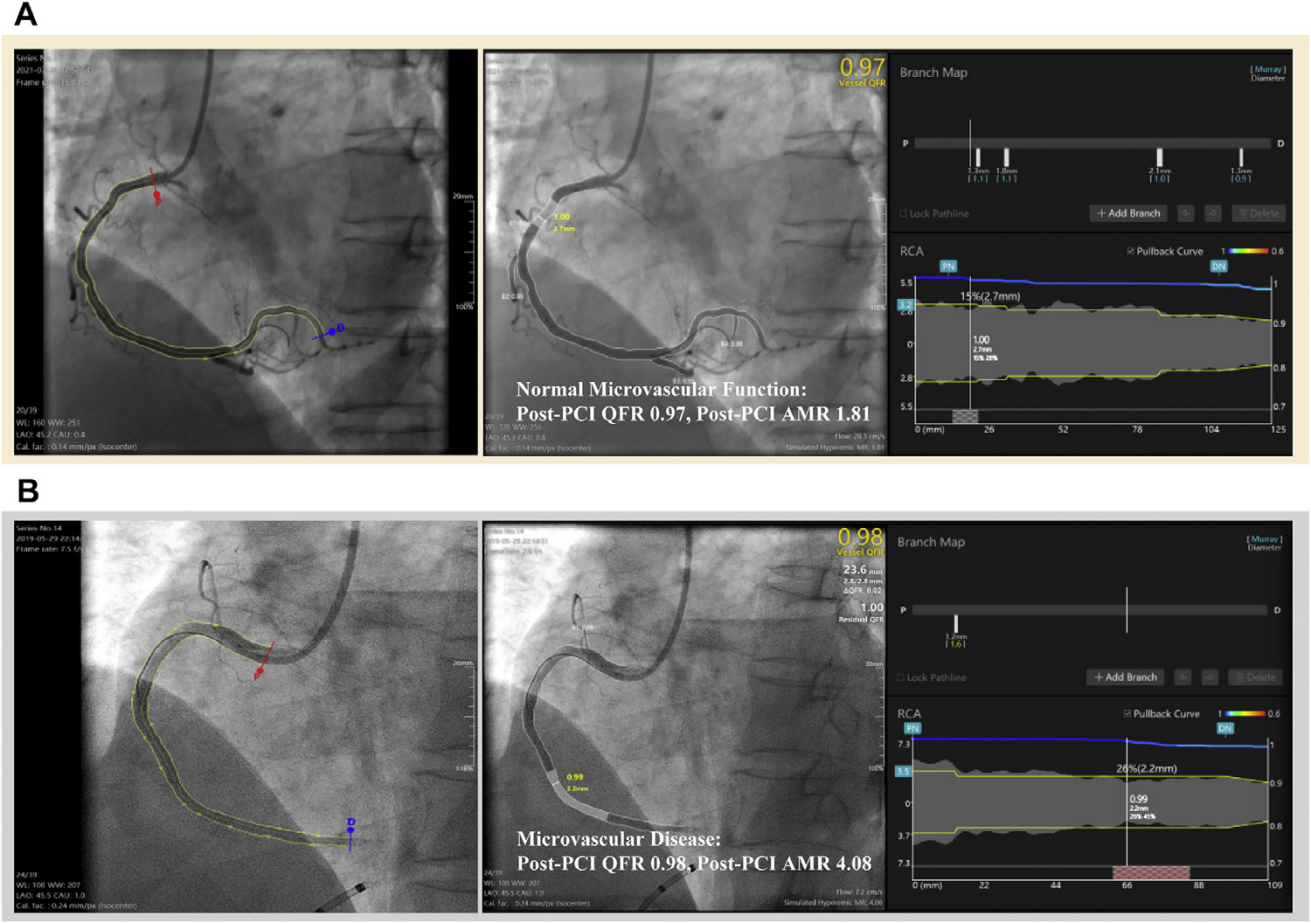
Derivation of angiography-derived physiological indices of coronary lesion in patients with STEMI.

In the RCS analysis, it was revealed the trend between the AMR and composite outcome. Initially, the risk of the endpoint event demonstrated a relatively stable pattern until approximately 2.55 mmHg*s/cm, after which it exhibited a notable rapid escalation (Fig. 2A). Patients were divided into different groups based on the most significant statistical differences at a cut-off value of 2.55 mmHg*s/cm, as shown in Fig. 2B. For example, we selected two angiographic images for analysis, finding that the measured AMR values respectively aligned with the low AMR (Fig. 3A) and high AMR (Fig. 3B) categories. In detail, 125 (53.9%) patients were classified into the high AMR group, while 107 (46.1%) patients had an AMR of less than 2.55 mmHg*s/cm. The baseline characteristics are presented in Table 1. The average door-to-wire time was 82.87±35.62 minutes, with no significant difference between the two groups. Additionally, 85.8% of the STEMI patients were male. In comparison to patients with low AMR, those with high AMR tend to be older in age. In the majority of instances, the IRA was identified as the Left Anterior Descending (LAD) coronary artery and 55.6% showed multivessel disease, which was characterized by stenosis over 50% in other coronary arteries in addition to the culprit vessels. The group with AMR ≥ 2.55 mmHg*s/cm showed a significant increase in post-PCI QFR compared to the group with AMR < 2.55 mmHg*s/cm. Neither group had significant differences in risk factors, discharge medicine, interventional procedure characteristics, or laboratory tests.

**Table 1:** Baseline characteristics.

	AMR < 2.55 mmHg*s/cm (n = 107)	AMR ≥ 2.55 mmHg*s/cm (n = 125)	p-value
**Admission**			
Age (year)	61.05 ± 10.86	66.03 ± 11.85	0.001
Male	95 (88.8%)	104 (83.2%)	0.305
Left ventricular ejection fraction (%)	55.96 ± 8.08	55.16 ± 9.70	0.501
Systolic blood pressure (mmHg)	131.34 ± 23.73	130.08 ± 22.14	0.677
Diastolic blood pressure (mmHg)	81.36 ± 15.40	78.66 ± 15.63	0.187
**Risk factors**			
Current smoker	66 (61.7%)	64 (51.2%)	0.141
Hypertension	57 (53.3%)	62 (49.6%)	0.67
Diabetes	32 (29.9%)	40 (32%)	0.841
Hyperlipidemia	41 (38.3%)	57 (45.6%)	0.324
Previous PCI	10 (9.3%)	4 (3.2%)	0.092
Chronic kidney disease	11 (10.3%)	17 (13.6%)	0.568
**Laboratory test**			
High-density lipoprotein cholesterol (mmoL/L)	1.10 ± 0.29	1.15 ± 0.31	0.238
Low-density lipoprotein cholesterol (mmoL/L)	2.92 ± 1.04	2.99 ± 0.96	0.598
Total cholesterol (mmoL/L)	4.93 ± 1.25	5.00 ± 1.21	0.667
Triglycerides (mmoL/L)	2.22 ± 1.77	2.15 ± 1.92	0.769
Non-high-density lipoprotein cholesterol (mmoL/L)	3.83 ± 1.23	3.86 ± 1.20	0.889
Glucose (mmoL/L)	8.75 ± 4.09	8.84 ± 3.68	0.869
eGFR (mL/min/m^2^)	92.25 ± 24.09	87.12 ± 23.33	0.101
Creatine (µmoL/L)	75.83 ± 23.94	80.21 ± 47.04	0.362
Uric acid (µmoL/L)	372.11 ± 94.99	364.15 ± 101.15	0.539
Urea (mmoL/L)	6.12 ± 1.99	6.39 ± 2.21	0.328
B-type natriuretic peptide (pg/mL)	218.73 ± 464.86	206.68 ± 408.58	0.834
HbA1c (%)	6.48 ± 1.28	6.53 ± 1.27	0.756
Hematocrit (%)	42.76 ± 4.45	42.52 ± 4.46	0.69
Neutrophil/White blood cell	0.76 ± 0.12	0.78 ± 0.11	0.191
**Discharge medication**			
Aspirin	105 (98.1%)	123 (98.4%)	1
P2Y12 inhibitor	107 (100%)	125 (100%)	1
Beta blocker	86 (80.4%)	90 (72%)	0.183
Statins	106 (99.1%)	118 (94.4%)	0.114
RAAS blockade	56 (52.3%)	65 (52%)	1
SGLT2i	14 (13.1%)	8 (6.4%)	0.132
**Operational characteristics**			
Door to wire time (min)	82.54 ± 38.56	83.15 ± 33.05	0.81
**Pre-PCI TIMI grade**			
0	84 (78.5%)	106 (84.8%)	0.448
I	20 (18.7%)	17 (13.6%)	
II	3 (2.8%)	2 (1.6%)	
**Post-PCI TIMI grade**			
II	0 (0%)	4 (3.2%)	0.174
III	107 (100%)	121 (96.8%)	
Trans-radial access	99 (92.5%)	114 (91.2%)	0.9
Glycoprotein IIb/IIIa inhibitor	20 (18.7%)	36 (28.8%)	0.101
Multivessel disease	66 (61.7%)	63 (50.4%)	0.111
**Infarcted related artery**			
LAD	62 (57.9%)	63 (50.4%)	0.045
LCX	5 (4.7%)	18 (14.4%)	
RCA	40 (37.4%)	44 (35.2%)	
Post-PCI QFR	0.89 ± 0.15	0.96 ± 0.03	<0.001

Values are mean ± SD or n (%). AMR, Angiographic Microvascular Resistance; PCI, Percutaneous Coronary Intervention; RAAS, Renin-Angiotensin-Aldosterone System; eGFR, Estimated Glomerular Filtration Rate; TIMI, Thrombolysis in Myocardial Infarction; SGLT2-I, Sodium-Glucose Cotransporter 2 Inhibitor; LAD, Left Anterior Desceding Artery; LCX, Left Circumflex Artery; RCA, Right Coronary Artery; QFR, Quantitative Flow Ratio.

### Prognostic value of AMR in patients with STEMI

**Fig. 4:**
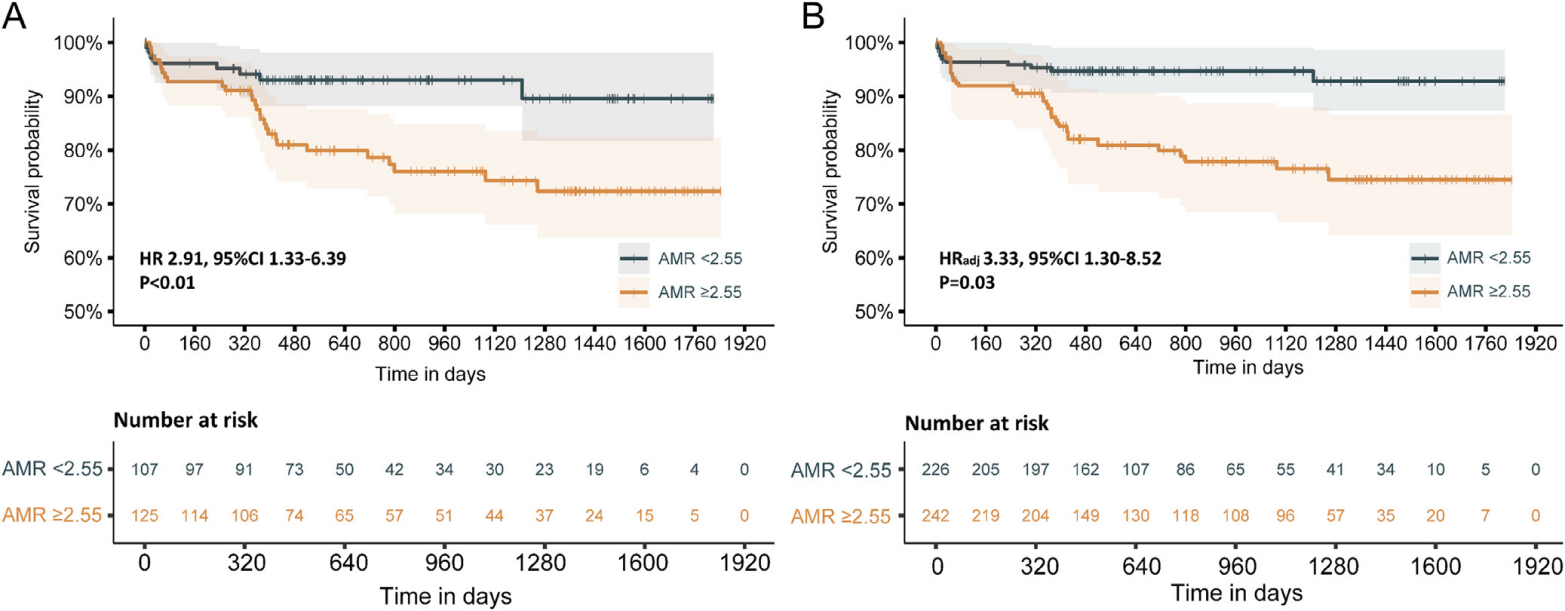
Kaplan Meier curves for all-cause death or readmission for heart failure according to AMR.

At a median follow-up of 1.74 years (range 1.07 to 3.65), 28 patients in the high-AMR group and 8 in the low-AMR group experienced the composite endpoint of all-cause death or heart failure readmission, as shown in Table 2. Cox proportional hazards analysis (Table 3) indicated that an AMR ≥ 2.55 mmHg*s/cm was an independent predictor of these events (HR = 2.33; 95% CI 1.04‒5.21; p = 0.04). Multivariate analysis identified AMR and Left Ventricular Ejection Fraction (LVEF%) as significant predictors of the composite outcome in STEMI patients. Kaplan-Meier survival curves showed that patients with an AMR ≥ 2.55 mmHg*s/cm had a significantly higher risk of all-cause mortality or readmission for heart failure compared to those with an AMR < 2.55 mmHg*s/cm (22.4% vs. 7.5%, HR=2.91; 95% CI 1.33‒6.39; p < 0.01) (Fig. 4A). After adjusting for age, gender, left ventricular ejection fraction, and post-PCI QFR using IPTW, the high-AMR group still exhibited a significantly higher risk for the composite outcome than the low-AMR group (22.4% vs. 7.5%, adjusted HR = 3.33; 95% CI 1.30‒8.52; p = 0.03) (Fig. 4B). Additionally, the patients with high-AMR owned a significantly elevated risk for readmission due to heart failure alone, compared to the low-AMR group (Table 2). There was no increased risk of myocardial infarction, revascularization, or readmission for unstable angina observed in either group during the follow-up period.

**Table 2:** Clinical Outcomes According to the stratification of AMR.

	Total (n = 232)	AMR < 2.55 mmHg*s/cm (n = 107)	AMR ≥ 2.55 mmHg*s/cm (n = 125)	p-value
**All-cause death or readmission for heart faliure**	36 (15.5%)	8 (7.5%)	28 (22.4%)	0.003
**All-cause death**	15 (6.5%)	4 (3.7%)	11 (8.8%)	0.195
**Cardiac death**	7 (3.0%)	2 (1.9%)	5 (4%)	0.575
**Readmission for heart failure**	21 (9.1%)	4 (3.7%)	17 (13.6%)	0.017
**Any myocardial infarction**	3 (1.3%)	0 (0%)	3 (2.4%)	0.303
**IRA myocardial infarction**	2 (0.9%)	0 (0%)	2 (1.6%)	0.547
**Non-IRA myocardial infarction**	1 (0.4%)	0 (0%)	1 (0.8%)	1
**Readmission for unstable angina**	17 (7.3%)	6 (5.6%)	11 (8.8%)	0.498
**Any revascularization**	11 (4.7%)	3 (2.8%)	8 (6.4%)	0.33

**Table 3:** Independent predictors for all-cause death or readmission for heart failure.

	HR (univariable)	p-value	HR (multivariable)	p-value
AMR ≥ 2.55 mmHg*s/cm	2.91 (1.33‒6.39)	<0.01	2.33 (1.04‒5.21)	0.04
**Left ventricular ejection fraction**	0.94 (0.91‒0.97)	<0.01	0.96 (0.93‒0.99)	0.01
**eGFR**	0.98 (0.97‒0.99)	<0.01	0.99 (0.97‒1.02)	0.57
**Creatine**	1.01 (1.00‒1.01)	<0.01	1.00 (1.00‒1.01)	0.33
**Urea**	1.17 (1.02‒1.33)	0.02	0.98 (0.80‒1.19)	0.81
**Chronic kidney disease**	2.68 (1.26‒5.70)	0.01	1.35 (0.37‒4.96)	0.65

AMR, Angiographic Microvascular Resistance; eGFR, Estimated Glomerular Filtration Rate.

### Prognostic nomogram development and performance

**Fig. 5:**
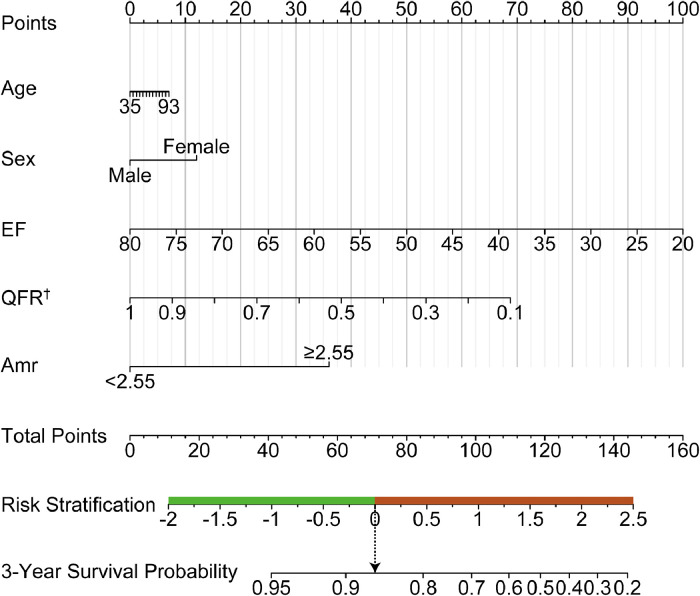
The prognostic nomogram uses AMR to measure the composite outcomes of STEMI patients after 3 years.

**Fig. 6:**
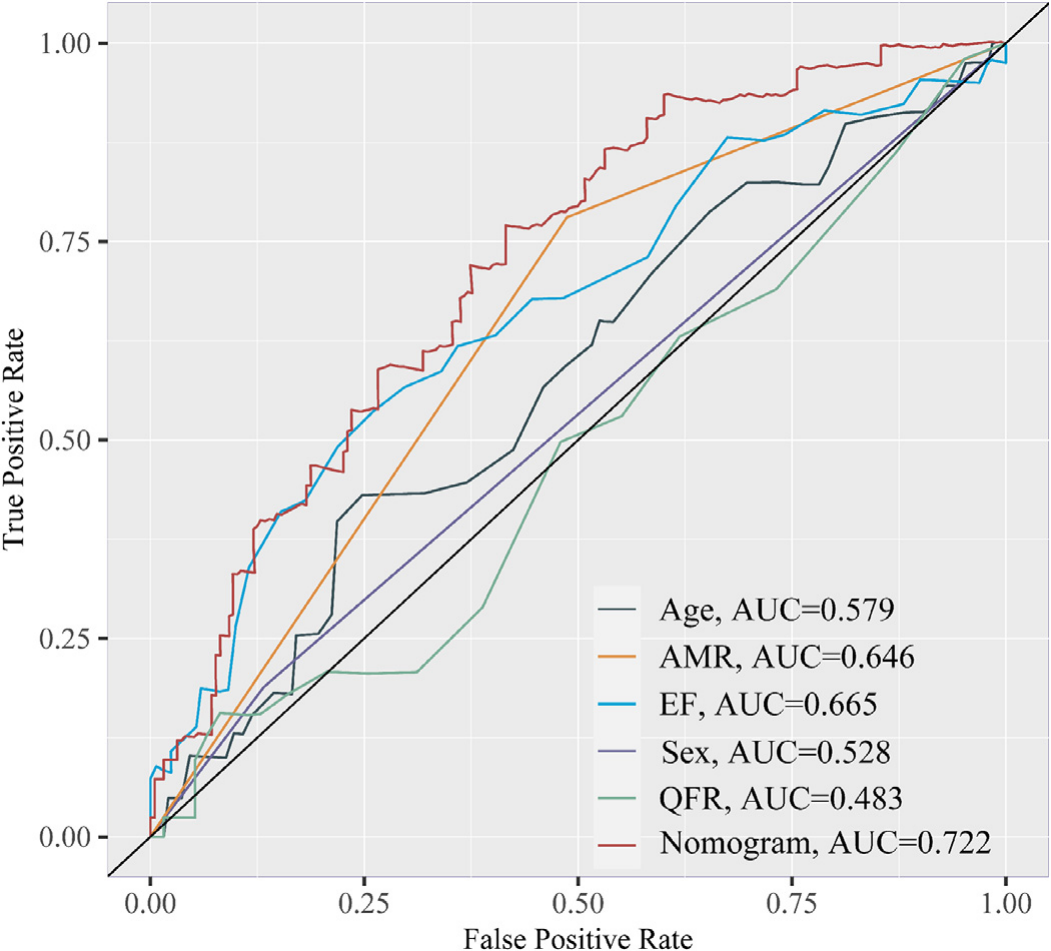
Receiver operating characteristic curves of AMR-involved nomogram over clinical factors.

**Fig. 7:**
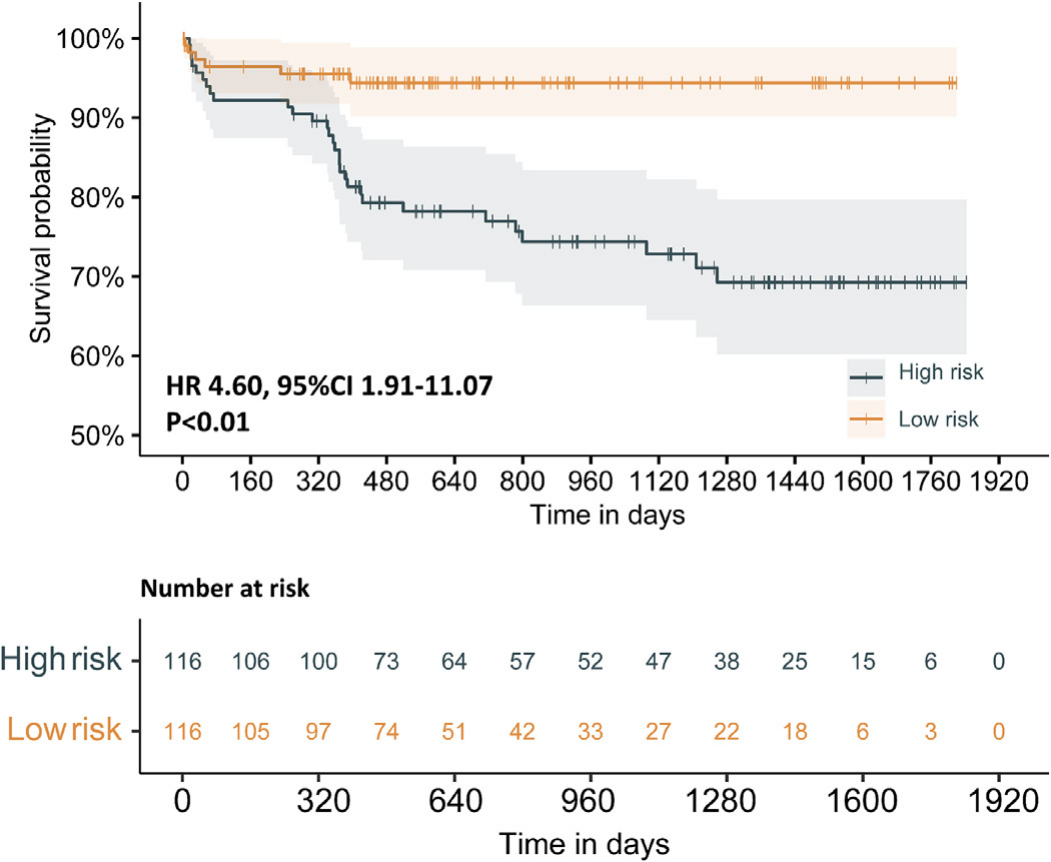
Kaplan Meier curves for all-cause death or readmission for heart failure according to risk stratification by nomogram.

After performing multivariate Cox analysis (Table 3 and Supplementary Fig. 1), AMR ≥ 2.55 mmHg*s/cm (p = 0.04), and left ventricular ejection fraction (p = 0.01) were found to be significant prognostic factors. Furthermore, we developed a prognostic nomogram based on demographics and coronary angiography-derived indices of coronary physiology, together with the variables mentioned above, for predicting STEMI-related survival probabilities at three years (Fig. 5). The performance of the nomogram is tested by the ROC curve (AUC = 0.722), better than age (AUC = 0.579), AMR (AUC = 0.646), LVEF (AUC = 0.665), sex (AUC = 0.528), post-PCI QFR (AUC = 0.483) alone (Fig. 6). Kaplan Meier survival curves also showed worse prognosis for patients with STEMI at 3 years in the high-risk group stratified by the nomogram (HR = 4.60; 95% CI 1.91‒11.07; p < 0.01) (Fig. 7). Moreover, the nomogram-derived high-risk population consists of 116 individuals with STEMI, 85.9% of whom will not suffer any composite endpoint events, compared to 94.4% of those at low risk.

### Access to the online AMR prediction tool

A digital calculator founded on the AMR model was effectively developed (see Supplementary Fig. 2), exhibiting superior performance compared to post-PCI AMR, post-PCI QFR, and various clinical parameters. This tool is accessible via a web-based platform at https://onepiece.shinyapps.io/amrnomo/. By incorporating 5 simple clinical variables, this tool could assist cardiologists in identifying STEMI patients who are at risk.

## Discussion

Studies have shown that even after restoring blood flow, nearly half of STEMI patients still experience adverse events as a result of CMD.^5^^,^^15^ In light of this, prompt and accurate evaluation of CMD is of utmost importance, especially during night shift emergency situations.^16^ We are also working to identify this category of patients at an early stage in order to prevent increased risk for mortality and other adverse events in the future.

Currently, Cardiac Magnetic Resonance (CMR) has traditionally been the representative of noninvasive imaging for the assessment of microcirculation in STEMI patients after primary PCI. CMR-detected Microvascular Obstruction (MVO), defined as hypotensive regions in the late gadolinium enhancement of CMR scans, was often used as a reference for CMD. Non-invasive imaging techniques, while invaluable, remain incapable of directly distinguishing between epicardial arteries and coronary microcirculation and are not widely used for the considerable cost and technique requirements. Furthermore, postoperative non-invasive imaging studies are often conducted in a relatively late phase, falling outside the therapeutic window for STEMI management.

During the procedure, former studies employed methods such as ST-Segment Resolution (STR),^17^ TIMI Myocardial Perfusion Grading (TMPG),^18^ TIMI Myocardial Perfusion Frame Count (TMPFC),^19^ and Myocardial Blush Grading (MBG)^18^^,^^20^ to provide real-time assessment of microcirculatory function in STEMI patients. Unfortunately, the poor reproducibility of these semi-quantitative indicators rendered them unsatisfactory in terms of precision and consistency. As endorsed by current guidelines,^21^ pressure wire and thermodilution-derived IMR offer quantitative and reproducible measurement since it is relatively specific for microcirculatory resistance, unaffected by epicardial coronary stenosis.^22^ The utilization of intra-coronary physiology allows real-time comprehension of the whole coronary circulation in conjunction with Fractional Flow Reserve (FFR) and Coronary Flow Reserve (CFR). Such insights empower timely interventions, exemplified by the intracoronary administration of nicorandil, which could reduce myocardial infarct size in patients with STEMI.^23^

However, the application of IMR is limited in clinical practice, especially in urgent conditions for some reasons, including the hyperemia-dependent setting and increased time and cost. During the 2000s, coronary physiology changed into the “era of subtraction” in response to the considerations mentioned above,^24^ non-hyperemic wire-derived pressure ratios showed the potential to surpass FFR. Furthermore, angiography-derived FFR has emerged in the 2010s.^25^, ^26^, ^27^, ^28^ Notably, the assessment of microcirculatory function followed a similar trajectory. Researchers^29^ validated IMRangio (Medis, Leiden, Netherlands), the first computational physiological index aimed at microcirculation. Using coronary angiography images from a steady hyperemic state, IMRangio demonstrated a strong correlation with IMR (*r* = 0.85, p < 0.001). An IMRangio value greater than 40U showed remarkable accuracy in identifying clinically significant CMD, validated by both IMR (p < 0.001) and CMR-based MVO (p = 0.006). To mitigate the adverse effects and limitations associated with hyperemic agents, the Non-Hyperemic angiography-derived Index of Microcirculatory Resistance (NH-IMRangio) was developed using a blood flow frame-based approach (Medis, Leiden, the Netherlands). NH-IMRangio also displayed a strong correlation with IMR (p < 0.0001) and CMR-detected MVO (p = 0.033) in STEMI patients.^30^ The OxAMI (Oxford Acute Myocardial Infarction) study recently highlighted NH-IMRangio's ability to predict long-term clinical outcomes in STEMI patients. The patients with NH-IMRangio value above 43U were faced with the elevated risk of all-cause mortality, resuscitated cardiac arrest, and heart failure (p = 0.047).^31^ Additionally, the coronary angiography-derived Index of Microcirculatory Resistance (caIMR) was developed (Rainmed Ltd., Suzhou, China).^32^ The calculation of caIMR requires two angiographic images at a 30° angle and real-time aortic pressure measurements, necessitating specialized sensor kits. A strong correlation was observed between caIMR and IMR (*r* = 0.746), with an accuracy of 84.2%, sensitivity of 86.1%, and specificity of 81.0%.^32^ Moreover, caIMR has shown significant diagnostic and prognostic value in STEMI patients undergoing emergency PCI, with a caIMR ≥ 40U identified as an independent risk factor for cardiac mortality and heart failure-related events during hospital admission.^33^ Recently, Wang et al.^34^ find that patients with higher caIMR exhibited less regression in infarct size and more persistent iron within the infarct zone at 3-month follow-up, indicating extensive and long-lasting microvascular impairment.

Compared to these angiography-derived indices mentioned above, AMR allows for a simpler approach since it only requires analysis based on a single angiographic view in less than one minute without the need for expensive pressure sensor kits (almost as expensive as pressure wire-based assessment). Theoretically, the computation of AMR depends on an estimated hyperemic velocity rather than the mean transit time that IMR used. To a certain extent, the variability of mean transit time has impacted the reproducibility of IMR in clinical practice.^35^ The absence of multi-positional imaging of a specific coronary artery poses challenges in obtaining measurements for some parameters mentioned above in many retrospective angiography studies. The single angiographic view-derived AMR further simplifies the feasibility of retrospective analysis. Clinical validation of AMR and IMR involving 163 patients and 257 vessels revealed a strong correlation between the two indices (*r* = 0.83, p < 0.01). In addition, inter-observer variability was also found to be minimal, underscoring the high reproducibility of AMR measurements.^10^ The formulation of AMR notably reveals its derivation through QFR computation. Our study finds that vessels with higher AMR have concordant QFR elevations, enhancing the robustness of the inferences drawn from these paired indices. Recent studies have indicated that high microvascular resistance, as denoted by a 3-vessel AMR value of ≥ 7.04, is linked to a poorer prognosis in patients with obstructive Hypertrophic Cardiomyopathy (oHCM).^36^ In this analysis, an Infarct-Related Artery (IRA) AMR of ≥ 2.55 mmHg*s/cm was significantly correlated with an increased risk of all-cause mortality or readmission for heart failure, throughout a median follow-up duration of 1.74 years.

As far as we know, our study expands the prognostic value of AMR in STEMI patients after successful PCI. The group with AMR ≥ 2.55 mmHg*s/cm consists largely of older individuals, similar to the finding of microvascular diseases which is consistent with the fact that the elderly are more likely to suffer from poorer vascular conditions.^37^ The multivariate Cox regression in this research also identified the LVEF value as an independent predictor of poor prognosis, in agreement with prior studies.^35^^,^^37^ Based on this study at a median 1.74 years of follow-up, an AMR ≥ 2.55 mmHg*s/cm was identified as an independent indicator of adverse events, approximately doubling the risk of negative clinical outcomes. While our dataset significantly uncovers a nexus between AMR and EF with events using diverse indicators, several other critical indices have also been observed to be connected. According to our study, 12.1% of our study population was found to be affected by chronic kidney disease, which is in line with a recent epidemiological study focusing on this group in China when the age of STEMI patients is considered.^38^ CKD-associated parameters (the estimated glomerular filtration rate and serum creatine) showed significance between the two groups in the univariable test but the multivariable test was not. Given that AMR is a parameter derived from QFR, we incorporated it alongside fundamental demographic parameters (age and gender) into the development of the nomogram. Significantly, it is also the first attempt to predict the incidence of events over the next three years among STEMI patients who have undergone emergency PCI through an AMR-infused nomogram. The model employing AMR exclusively has an AUC value of 0.646, comparable to EF (AUC = 0.665) and higher than models solely based on age, sex, or post-PCI QFR. Furthermore, by combining multiple indicators, including AMR, diagnostic efficacy has reached a satisfactory level. Moreover, the nomogram-derived high-risk population showed worse prognosis for patients with STEMI at 3 years. (HR = 4.60; 95% CI 1.91‒11.07; p < 0.01), supporting the role of angiography-derived physiological indices in outcome prediction and risk stratification in STEMI patients. Therefore, AMR has emerged as a convenient, safe, and cost-effective evaluation method. Our study indicates that AMR has the potential to be a feasible alternative for IMR and become the preferred method for assessing coronary microvascular function in the future, especially in the setting when hyperemic agents and further wire-based assessment are limited.

### Limitation

Our current study is subject to several limitations. First, an inherent limitation may introduce biases into our results because of its single-center, retrospective nature. Further, the relatively small sample size and short follow-up duration have limited the number of cardiovascular events observed. Testing in larger external cohorts is therefore recommended for further validation and improvement of our results.

Additionally, invasive measurement of IMR was not performed on the study population, making it difficult to compare and validate IMR and AMR in a single-center setting. The larger scale of multicenter, real-time computation of AMR will address this issue through prospective considerations in the future. Although we took a first look at the relationship between AMR and clinical outcomes in STEMI patients, image tests should be included in the future study to gain a better understanding of STEMI imaging findings. For analysis, angiographic images must be of high quality with appropriate angles for imaging so this study excluded certain high-risk patients in clinical practice, such as those with complex coronary structures, which may have led to some discrepancy between our research and real-world clinical outcomes.

## Conclusion

STEMI patients after successful PCI whose IRA's AMR ≥ 2.55 mmHg*s/cm exhibited a significantly higher risk of all-cause mortality or readmission for heart failure. The AMR-involved nomogram is an appropriate risk stratification tool for STEMI patients after primary PCI.

## Ethics statement

The Ethical Committee of the Southwest Hospital (B) KY2023069 provided its endorsement on June 20, 2023. The requirement for informed consent was exempted.

## Authors’ contributions

Conceptualization: Z Zhang and X Qu; Data curation: G Qian, H Qin, Y Feng, D Deng and C Zhang; Formal analysis: G Qian, H Qin and Z Zhang; Funding acquisition: Z Zhang; Investigation: G Qian, H Qin, D Deng; Methodology: G Qian, X Qu and Z Zhang; Resources: Z Zhang and X Qu; Supervision: Z Zhang and X Qu; Validation: D Deng, Y Feng and C Zhang; Visualization: G Qian; Roles/Writing - original draft: G Qian; and Writing-review & editing: G Qian and Z Zhang. Every author has reviewed and given their approval to the final version of the manuscript.

## Funding

This study was completed with the fund of the Chongqing Science and Technology Bureau (Grant numbers [CSTB2023TIAD-KPX0061-1]).

## Declaration of competing interest

The authors declare no conflicts of interest.

## Data Availability

Not available.

## Appendix Supplementary materials

Image, application 1
