# Supplementary material for: Prognostic value of angiographic microvascular resistance in patients with ST-segment elevation myocardial infarction
Source: Clinics (Sao Paulo). 2024 Jul 24;79:100429. doi: 10.1016/j.clinsp.2024.100429 (PMC11327545; doi:10.1016/j.clinsp.2024.100429)

CLINICS-D-24-00262_Supplementary Material

**Supplementary Figure 1** The Forest plot of the multivariate Cox proportional hazards regression analysis.


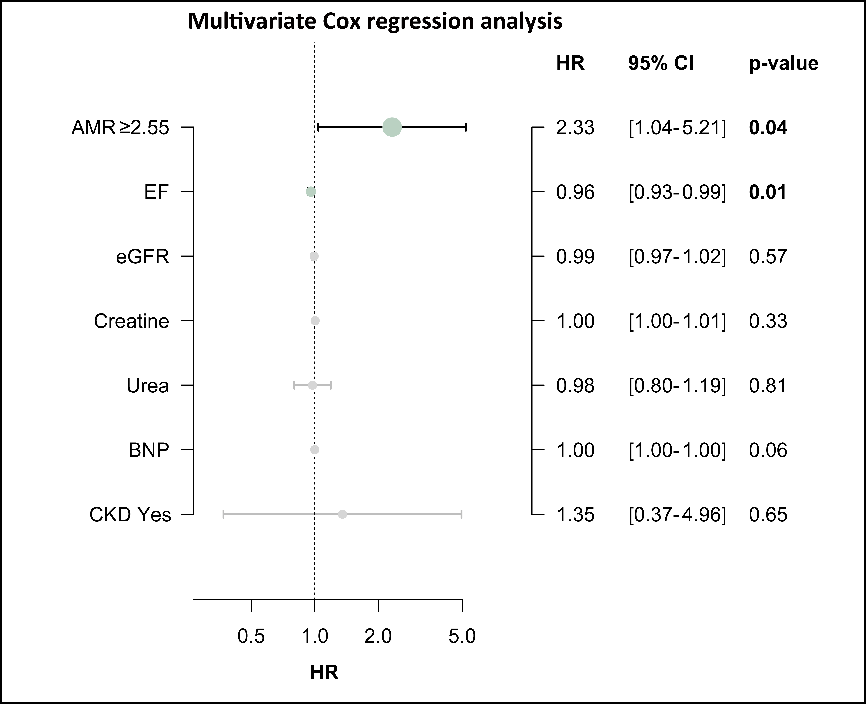


**Supplementary Figure 2** Free calculator based on the AMR model, available on the web.


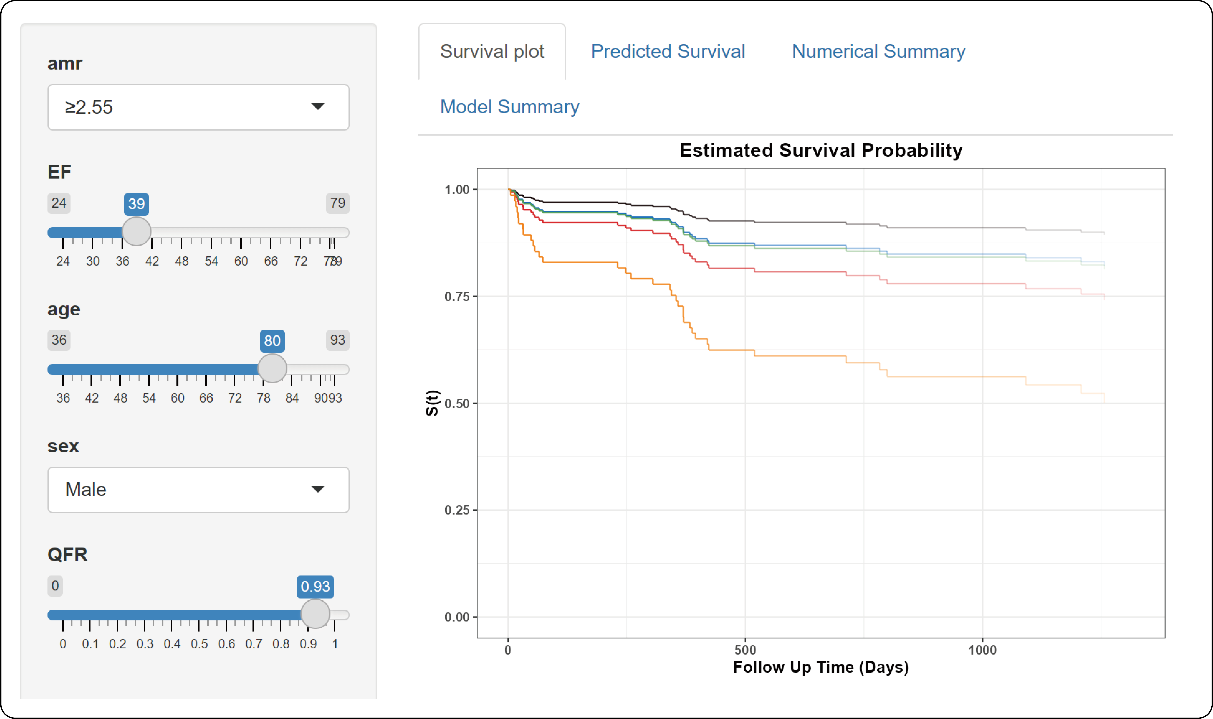

Supplement: Supplementary file 1 [file mmc1.docx]
